# Evolutionary Conservation of Infection-Induced Cell Death Inhibition among *Chlamydiales*

**DOI:** 10.1371/journal.pone.0022528

**Published:** 2011-07-22

**Authors:** Karthika Karunakaran, Adrian Mehlitz, Thomas Rudel

**Affiliations:** Department of Microbiology, Biocenter, University of Würzburg, Würzburg, Germany; Indian Institute of Science, India

## Abstract

Control of host cell death is of paramount importance for the survival and replication of obligate intracellular bacteria. Among these, human pathogenic *Chlamydia* induces the inhibition of apoptosis in a variety of different host cells by directly interfering with cell death signaling. However, the evolutionary conservation of cell death regulation has not been investigated in the order *Chlamydiales*, which also includes *Chlamydia*-like organisms with a broader host spectrum. Here, we investigated the apoptotic response of human cells infected with the *Chlamydia*-like organism *Simkania negevensis* (Sn). *Simkania* infected cells exhibited strong resistance to apoptosis induced by intrinsic stress or by the activation of cell death receptors. Apoptotic signaling was blocked upstream of mitochondria since Bax translocation, Bax and Bak oligomerisation and cytochrome *c* release were absent in these cells. Infected cells turned on pro-survival pathways like cellular Inhibitor of Apoptosis Protein 2 (cIAP-2) and the Akt/PI3K pathway. Blocking any of these inhibitory pathways sensitized infected host cell towards apoptosis induction, demonstrating their role in infection-induced apoptosis resistance. Our data support the hypothesis of evolutionary conserved signaling pathways to apoptosis resistance as common denominators in the order *Chlamydiales*.

## Introduction

*Simkania negevensis* (Sn) is a *Chlamydia*-like organism [1] belonging to the family *Simkaniaceae*, order *Chlamydiales* [2]. They are obligate intracellular gram-negative bacteria which replicate within endocytic vacuoles of eukaryotic cells i.e. amoebae, human epithelial cells and macrophages [3]. *Simkania* has been reported as an emerging pathogen associated with several types of respiratory tract infection such as bronchiolitis in infants [4], [5], [6], [7], community acquired pneumonia [8], [9], [10], chronic obstructive pulmonary disease in adults [11] and acute rejection in lung transplant recipients [12]. Moreover, seroprevalence rates in adults between 46–80% suggest a broad distribution of the organism in human populations [13].

*Simkania* replicates in a biphasic life cycle similar to *Chlamydia*. Infection starts with an electron dense elementary body which differentiates into the electron lucent replicative form called the reticulate body. The active replication cycle of Sn takes about 3–5 days and extensive long term relationship with the host cell of about 10–15 days has been reported [14]. The strategy that this pathogen takes to keep its host alive for such a long period of time is unknown.

Over the past two decades, a substantial body of evidence has pointed out the importance of apoptosis, a form of programmed cell death as an evolutionary conserved strategy for host defense against bacterial and viral infections [15]. Pathogens in response utilize idiosyncratic mechanisms to modulate host cell apoptotic pathways [16]. *Neisseria gonorrhoeae* and *Staphylococcus aureus* induce apoptosis in non-phagocytic host cells [17], [18] while bacteria like *Salmonella spp.* and *Shigella flexneri* trigger cell death in host macrophages [19], [20]. Destruction of epithelia and/or depletion of immune cells, respectively, may help the pathogen to invade and spread out to deeper tissues of an infected host. Induction of apoptosis by *Chlamydia* and *Parachlamydia* has also been observed [21], [22], however, it is generally accepted and experimentally verified, that obligate intracellular pathogens like *Chlamydia* and *Rickettsia* confer resistance to apoptosis induced by infection stress or by external stimuli [23], [24]. This serves as a pathogenic strategy evolved to promote a long-term relationship to complete the replication cycle and survival in the host cell.

Induction of apoptosis involves a cascade of molecular events organized in two major pathways: the extrinsic or the death receptor pathway and the intrinsic or mitochondrial pathway. The main effectors in both the pathways are a family of cysteinyl aspartate directed proteases (caspases) that cleave a wide range of cellular death substrates which ultimately results in apoptotic cell death [25]. Whereas the extrinsic pathway is initiated by ligand-dependent activation of cell death receptors, diverse stimuli like anticancer drugs or cellular stress trigger the intrinsic pathway. Mitochondria are the central regulators of intrinsic apoptotic pathways by integrating diverse pro- and antiapoptotic signals. Apoptotic signals initiate the opening of the outer mitochondrial membrane (OMM) and the release of caspase-activating molecules like cytochrome *c* into the cytoplasm [26]. The family of Bcl-2 proteins plays a pivotal role in controlling the integrity of the OMM. The so-called BH3-only proteins subfamily are stress sentinels which induce the multimerization of Bak and Bax proteins and the formation of pores in the OMM. Inhibitory Bcl-2 family members like Bcl-2 and Mcl-1 keep Bak and Bax in their monomeric inactive form and thereby prevent cytochrome *c* release and apoptosis induction [27], [28].

The activity of caspases can be fine tuned by the direct binding of *X-linked* inhibitor *of apoptosis protein* (XIAP) [29], [30]. XIAP and other members of the *inhibitor of apoptosis* (IAP) family like cIAP-1 and cIAP-2 possess E3-ligase activity involved in controlling the stability of apoptosis regulatory pathways [31]. The phosphoinositide 3-kinase (PI3K)-mediated activation of protein kinase B (Akt) and Raf-MEK-ERK MAP-kinase pathway have emerged as an important pathway involved in regulating the signaling of cell proliferation, survival and anti-apoptosis [32], [33], [34]. These pathways control apoptosis not only at the transcriptional, but also at the post translational level. For example, Raf and Akt inactivate Bcl-2-associated death promoter (Bad), a BH3 only pro-apoptotic protein by phosphorylation [35], [36].

The mechanism underlying apoptosis resistance of cells infected with *C. trachomatis* and *C. pneumoniae* have been intensively investigated (for review see [37]). *Chlamydia*-infected cells are protected against a wide variety of cell death inducers including death receptor agonists, anticancer drugs, and double-stranded RNA reflecting virus-induces cell death [23], [38], [39], [40], [41]. *Chlamydia* infection induces the induction and/or stabilization of cIAP-1, cIAP-2, XIAP and Mcl-1 [41], [42], [43], [44]. Moreover, BH3-only family members have been demonstrated to be degraded [45], [46] or recruited to inclusions [47] in infected cells. Most of these studies came to the conclusion, that infection blocks apoptosis at or upstream of the mitochondria.

This study focuses on the evolutionary conservation of cell death inhibition among members of *Chlamydiales*. We found that Sn-infected cells strongly resist apoptosis induced by various external stimuli. Pathways subverted in Sn-infected cells to prevent apoptosis were similar, but not identical to those modulated by *Chlamydia* or *Chlamydophila*. Our data suggest an evolutionary conservation of mechanism underlying apoptosis regulation in distantly related members of the order *Chlamydiales*.

## Results

### S. negevensis infected cells are resistant to apoptosis

**Figure 1:**
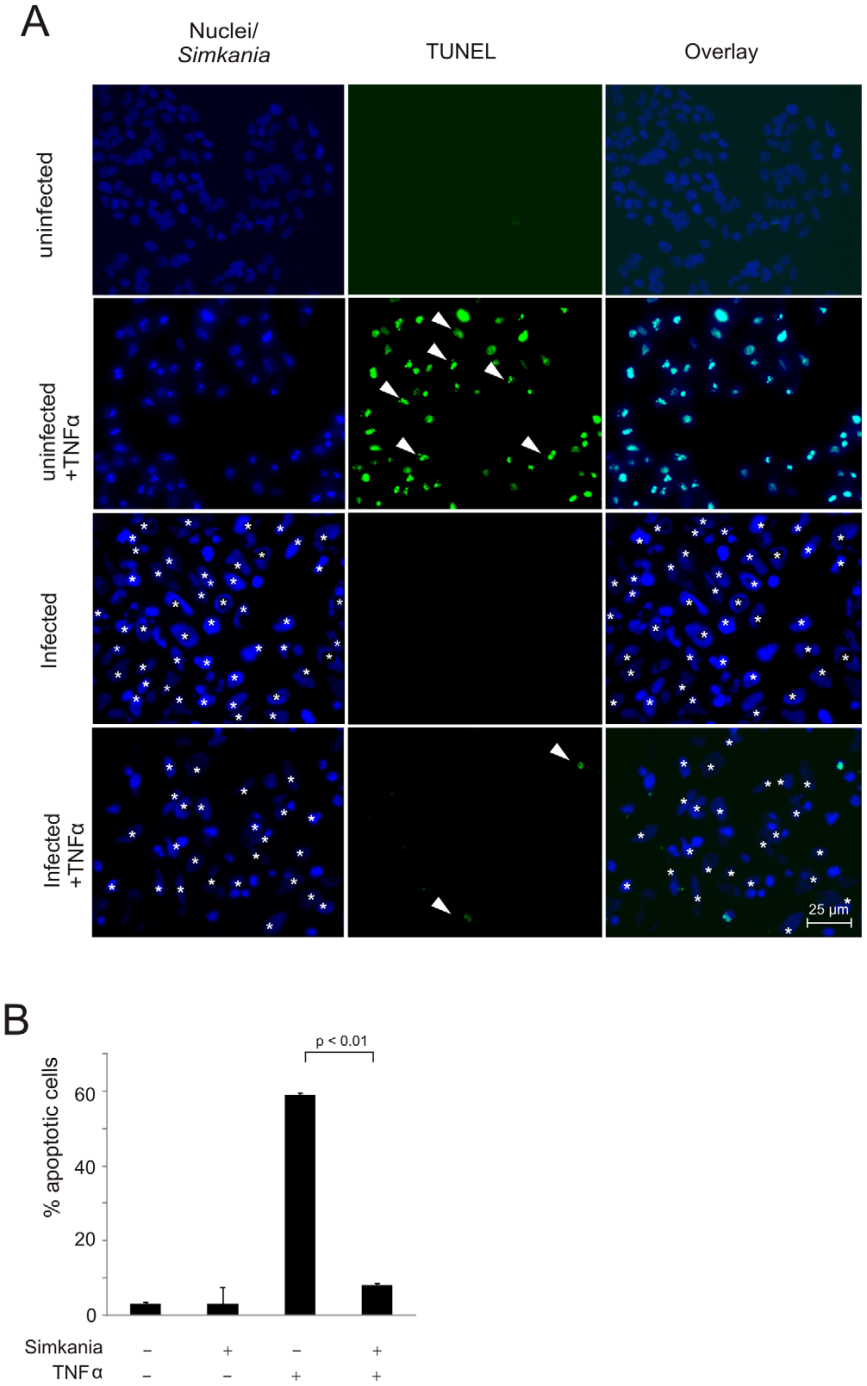
*Simkania negevensis* infected HeLa cells are resistant to apoptosis induced by TNF-α. (**A**) HeLa cells with or without *Simkania* infection (MOI 1) were treated with 20 ng/ml TNF-α+3 µg/ml Chx or with carrier for 4 hours. Samples were stained with Hoechst (blue) and TUNEL (green) and viewed under a fluorescent microscope. The infected cells are marked by white asterisk. White arrowheads mark example apoptotic cells. n = 2 (**B**) Bar diagram displaying the quantitative analysis of the experiment shown in Figure 1A. Cells from five random fields were counted under a 40× objective and the percentage of apoptotic cells was calculated. *Simkania* infection significantly decreased the number of apoptotic cells upon TNF-α stimulation to nearly background levels (n = 2, error bars = SE).

**Figure 2:**
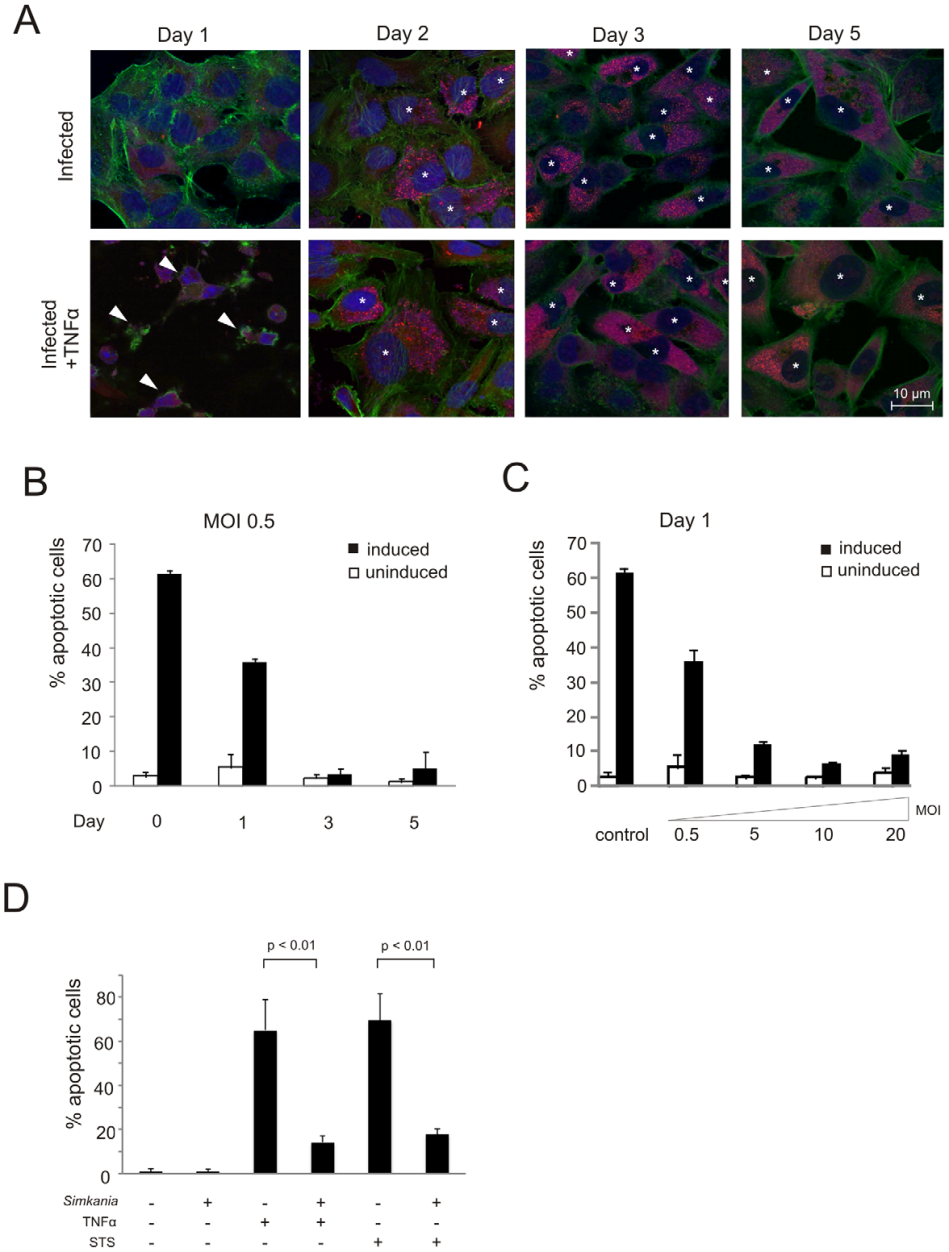
Apoptosis inhibition by *Simkania* is a function of time and infection dose and is independent of the stimulus. (**A**) Immunofluorescence analysis of the time and dose dependency of *Simkania* mediated anti-apoptosis. HeLa cells were infected with *Simkania* for 1–5 days, MOIs 0.5, 5.0, 10 and 20, induced with 20 ng/ml TNF-α+3 µg/ml Chx or with carrier for 4 hours and stained with Draq5 (nuclei+inclusions, blue), Syto82 (accumulates in *Simkania*, red) and Phalloidin (actin, green). The infected cells are marked by white asterisk. Images shown are from an experiment with MOI 0.5; MOIs 5, 10 and 20 are not shown. Apoptotic cells are marked by white arrowheads. Infection led to apoptosis resistance from day 1 on. n = 3 (**B**) Bar diagram displaying the quantitative analysis of the time dependency at MOI 0.5 from the experiment shown in Figure 2A. Cells from five random fields were counted under a 40× objective and the percentage of apoptotic cells was calculated. Apoptosis resistance increased till day 3; towards the end of the infection cycle apoptosis sensitivity slightly increased until day 5. At higher MOIs infected cells became more susceptible to apoptosis towards end cycle (data not shown). n = 3, error bars = SE (**C**) Bar diagram displaying the quantitative analysis of the MOI dependency at day 1 from the experiment shown in Figure 2A. Quantification as described in Figure 2B. Apoptosis resistance was found to be clearly dose dependent, increasing to MOI 10. At MOI 20 increased apoptosis sensitivity was observed. n = 3, error bars = SD (**D**) Bar diagram displaying the quantitative analysis of an infection/apoptosis induction experiment using either TNF-α or STS. HeLa cells with or without *Simkania* infection (MOI 0.5) were treated with 20 ng/ml TNF-α+3 µg/ml Chx, STS 1 µg/ml or with carrier for 4 hours. Samples were stained with Hoechst 33258 (images not shown), five random fields were counted under a 40× objective and percentage of apoptotic cells was calculated. *Simkania* infection significantly decreased the number of apoptotic cells upon TNF-α or STS stimulation (n = 2, error bars = SE).

Apoptosis resistance is a hallmark of *Chlamydia* and *Chlamydophila* infection but has so far not been investigated for other family level related bacteria like *Simkania negevensis*. To test whether *Simkania* confers apoptosis resistance during infection, we induced apoptosis with tumor necrosis factor alpha (TNF-α) in the presence of cycloheximide (TNF/Chx) in infected and non-infected HeLa cells. The cells were fixed and stained with Hoechst 33258 to detect chromatin condensation (Figure S1) and apoptotic cells were in addition quantified by terminal transferase mediated dUTP nick end labeling (TUNEL). A nearly complete block of apoptosis induced by TNF-α was observed at day 3 post infection (p.i.) (Figure 1A, S1) illustrated by a reduction in the number of apoptotic cells from ∼60% to ∼10% in infected cells (Figure 1B). These results suggested an anti-apoptotic activity in cells infected with *Simkania*. Time course experiments showed time-dependent resistance for TNF/Chx-induced apoptosis at a multiplicity of infection (MOI) of 0.5 beginning at day 1 p.i. and was similar to non-treated cells at day 3 with <10% apoptotic cells (Figure 2A, B). Further, apoptosis resistance was found to be dependent on the MOI; cells infected with higher MOI acquired apoptosis resistance much faster as demonstrated in a quantification of TNF/Chx-induced apoptosis at day 1 using MOIs ranging from 0.5 to 20 (Figure 2C).

Since the massive infection may directly affect signaling via the tumor necrosis factor receptor 1 (TNFR1), we determine general signaling of this receptor during infection by stimulation with TNF-α in the absence of Chx. This treatment resulted in phosphorylation of MEK and ERK [48] in both non-infected as well as infected cells (Figure S2), demonstrating intact TNFR1 signaling.

To test whether the intrinsic apoptosis pathway is inhibited, *Simkania*-infected cells were treated with the broad range kinase inhibitor Staurosporine (STS). Infection reduced the apoptotic population in STS-treated cells in the same manner as observed in TNF/Chx-treated cells (Figure 2D). Taken together, *S.negevensis* inhibits both TNF/Chx and STS-induced apoptosis in a time and infection dose-dependent manner.

### Caspases are inhibited in Simkania-infected cells

**Figure 3:**
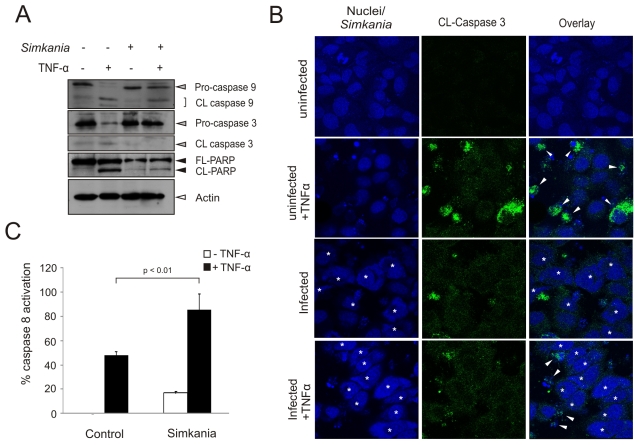
Differential caspase activation during *Simkania* infection. HeLa cells with or without *Simkania* infection (MOI 0.5, 3 days) were treated with 20 ng/ml TNF-α+3 µg/ml Chx for 4 hours before analysis. (**A**) Immunoblot showing the activation status of caspases-3, -8 and -9 (grey arrowheads) as well as PARP (black arrowheads) cleavage. Caspase-9 and -3 were not found to be cleaved in infected cells compared to uninfected induced cells. PARP is also not cleaved nor activated on induction in *Simkania* infected cells. Actin (white arrowhead) is used as the loading control. n = 2 (**B**) Immunofluorescence analysis showing that caspase-3 is not activated in *Simkania* infected cells on induction with TNF-α. Nuclei and bacteria were stained with Draq5 (blue) and activated caspase-3 was detected with an antibody specific for the cleaved protease (green, CL-Caspase 3). Infected cells are marked by white asterisk, white arrowheads mark active caspase-3. Bacterial inclusions are exemplified by yellow open and nuclei by red filled circles. n = 2 (**C**) Bar diagram showing the result of a caspase-8 activity assay (caspase-8 glo). Activation of caspase-8 is not blocked by *Simkania* infection. n = 3, error bars = SE.

TNF-α-induced apoptosis depends on a well characterized signaling cascade [49] and is thus suited to more precisely define the block in apoptotic signaling in *Simkania*-infected cells. To investigate whether *Simkania* blocks the proteolytic cleavage and activation of caspases, infected and TNF/Chx-treated cells were tested for caspase processing and activity. Both caspase-3 cleavage and activity were inhibited by *Simkania* infection (Figure 3A and B). Immunostaining revealed that the infected cells showed only ∼11% of activated caspase-3 against ∼35% of the uninfected induced cells (Figure S3). Caspase-3 cleavage leads to proteolytic degradation of key proteins such as the nuclear enzyme poly-ADP ribose polymerase (PARP). We found that PARP degradation was inhibited in *Simkania*-infected TNF/Chx-induced cells (Figure 3A), confirming a strongly reduced caspase-3 activity in infected cells. We then tested if infection also affects signaling upstream of the executioner caspase i.e. caspase-9 and caspase-8 processing or activity, respectively. Interestingly, while caspase-9 processing was blocked, caspase-8 activity was even increased by infection with *Simkania* (Figure 3A and C), demonstrating the selective inhibition of caspases in cells infected with *Simkania*.

### Simkania infection prevents cytochrome c release and Bax/Bak activation

**Figure 4:**
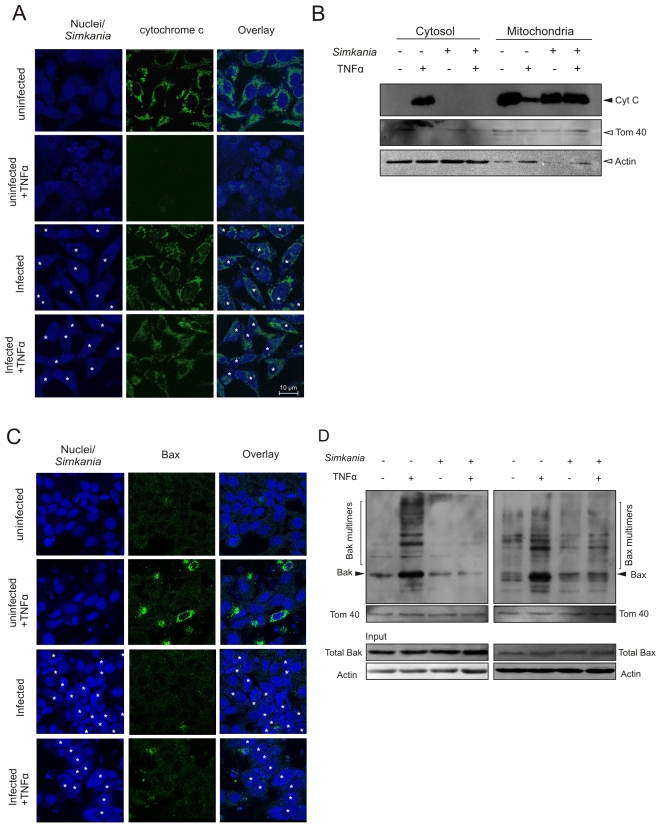
Bax/Bak activation and cytochrome *c* release. (**A**) Immunofluorescence staining of the effect of *Simkania* infection on cytochrome *c* distribution. HeLa cells with or without *Simkania* infection (MOI 1, 3 days) were treated with 20 ng/ml TNF-α+3 µg/ml Chx or carrier for 4 hours before analysis. Cells were stained for cytochrome *c* (green), *Simkania*/nuclei (blue) and viewed with a confocal microscope. Upon apoptosis induction cytochrome *c* is released from the mitochondria (uninfected+TNF-α), this was not the case for infected cells (infected+TNF-α). The infected cells are marked by white asterisk, n = 2 (**B**) Western blot analysis for cytochrome *c* release. Cytosol and mitochondria were isolated as described in the experimental procedures section and cytochrome *c* was detected by Western blot. Cytochrome *c* release was completely blocked by *Simkania* infection. Tom40 was used as a marker for mitochondria and Actin as a marker for cytosol. (**C**) Immunofluorescence staining of active Bax with a conformation sensitive antibody [65] after *Simkania* infection. HeLa cells were treated as described in Figure 4A. Images show mitochondrial localization of active Bax (green) in control-induced cells. Bax was not activated in TNF treated *Simkania* infected cells. The infected cells are marked by white asterisk. n = 2 (**D**) Immunoblot analysis of Bax and Bak after mitochondrial isolation and *in vitro* cross linking. HeLa cells with or without *Simkania* infection (MOI 1, 3 days) were treated with 20 ng/ml TNF-α+3 µg/ml Chx or carrier for 4 hours before mitochondria isolation, *in vitro* cross linking and analysis. Bax and Bak (black arrows) are not activated in *Simkania* infected induced cells as shown by background Bax and Bak oligomerisation (brackets). Equal loading was verified by Bradford assay and Tom40 western blot. Input Bak, Bax and Actin showed no differences in expression or loading. n = 2.

Block of caspase-9/-3 in the presence of active caspase-8 is indicative of an apoptosis inhibition at or upstream of the mitochondria [50]. Mitochondrial cytochrome *c* is translocated to the cytosol of cells undergoing apoptosis where it participates in caspase-9 activation [51]. Hence, we tested if Sn infection can prevent cytochrome *c* release from the cells treated with TNF/Chx induction. While TNF/Chx treatment induced the release of cytochrome *c* from mitochondria of uninfected cells, this was not the case in *Simkania*-infected cells (Figure 4A, B). Failure to release cytochrome *c* indicated incomplete activation of Bax and/or Bak upon apoptosis induction. Bax, once activated undergoes a conformational change permitting insertion into the mitochondrial membranes. First, we excluded that either Bax or Bak are differentially transcribed during infection (data not shown). We then tested whether Bax translocation into mitochondria is affected in Sn-infected cells upon TNF/Chx-induced apoptosis. Immunostaining confirmed strongly reduced levels of translocated Bax in infected cells upon apoptosis induction (Figure 4C). Once activated Bax and Bak form oligomers in the mitochondrial outer membrane leading to the release of pro-apoptotic factors like cytochrome *c*. We tested Bax and Bak oligomerisation via mitochondria isolation and *in vitro* cross-linking. Neither Bax nor Bak were found to form oligomers after TNF-α treatment in *Simkania*-infected cells in comparison to non-infected cells (Figure 4D). Thus, *Simkania* infection inhibits Bax translocation to the mitochondria as well as Bax/Bak oligomerisation.

### Bcl-2 family proteins are not differentially regulated in Simkania infection

**Figure 5:**
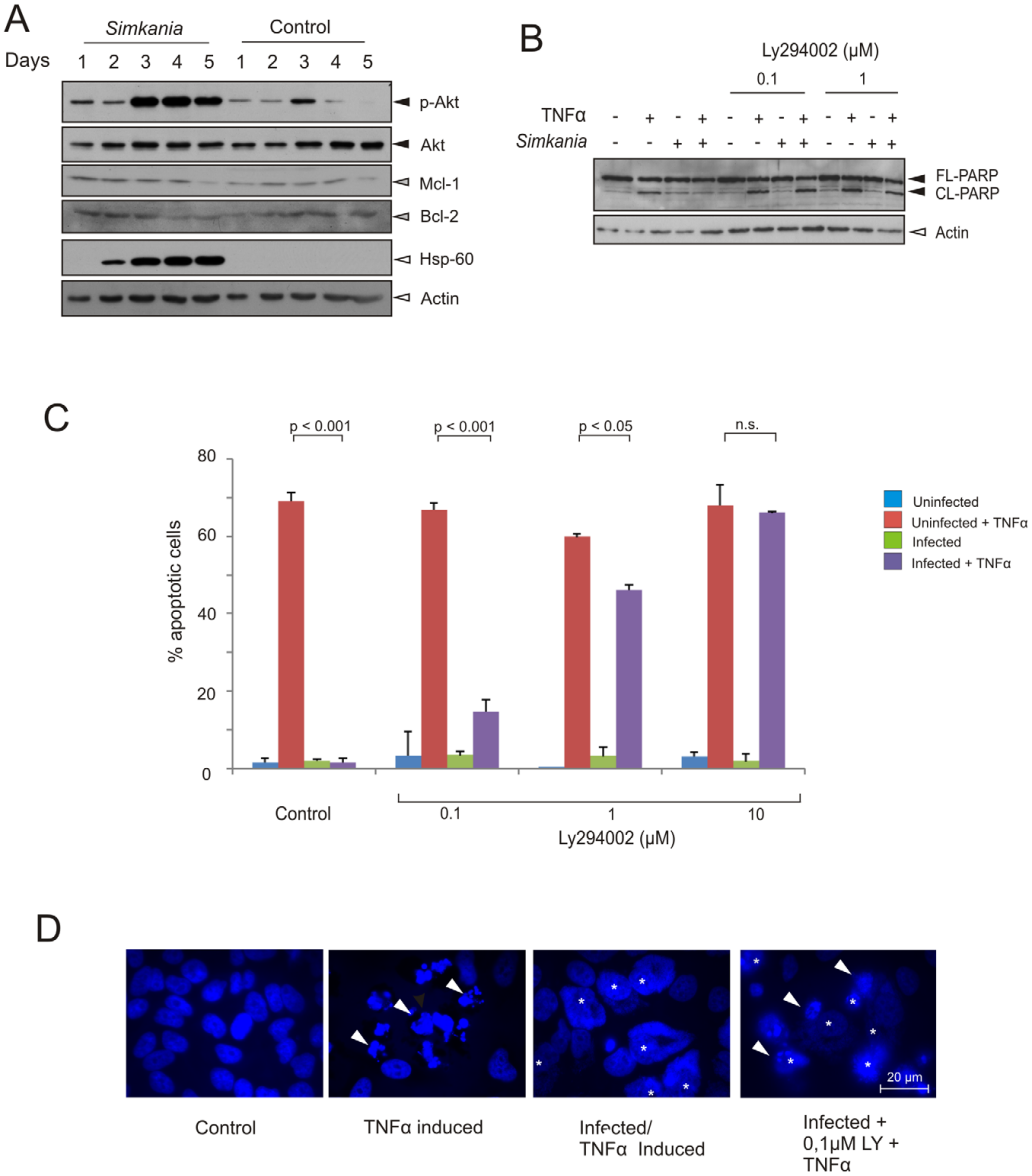
Regulation of Bcl-2 family members and PI3 kinase signaling during *Simkania* infection. (**A**) Immunoblot analysis of the anti-apoptotic/pro-survival signaling during S*imkania* infection. HeLa cells were infected with *Simkania* or Mock (control) in a time course experiment before analysis. Anti-apoptotic Bcl-2 family members like Mcl-1 or Bcl-2 (grey arrowheads) are not regulated. Akt is strongly activated during infection (black arrowheads). Hsp-60 and Actin (white arrowheads) were used as loading controls. n = 2. (**B**) Immunoblot analysis of PARP cleavage in S*imkania* infected cells after treatment with PI3K inhibitor. S*imkania* infected HeLa 229 cells were treated with Ly294002 (PI3 kinase Inhibitor) on day 3 post infection for 6 hours and induced with 20 ng/ml TNF-α+3 µg/ml Chx or carrier for 4 hours before analysis. PARP cleavage (black arrowheads) was a measure of apoptosis sensitization. Treatment with 0.1 µM LY29004 clearly sensitized infected cells to apoptosis. Hsp-60 and Actin (white arrowhead) were used as loading controls. n = 2. (**C**) Bar diagram showing samples treated similar as in Figure 5B but prepared for immunofluorescence analysis. Hoechst stained cells were counted to determine the number of apoptotic cells (40× magnification, five fields per sample, n = 2, Error bars = SE). 1 µM LY29004 was found to restore apoptosis sensitivity to ∼70% of control (**D**) Immunofluorescence of samples treated as described in Figure 5B–C. Samples were stained with Hoechst (blue) and viewed under a fluorescent microscope for counting. Hoechst dye stained both HeLa cell nuclei and *Simkania* inclusions (yellow mark). White arrowheads mark example apoptotic cells. n = 2.

Resistance of apoptotic signaling upstream of mitochondria has previously been shown to involve Bcl-2 family members in *Chlamydia*-infected cells. One mechanism involves the degradation of pro-apoptotic BH3-only proteins acting to either directly or indirectly induce Bak and/or Bax oligomerisation and mitochondrial outer membrane permeabilization [46], [52]. We tested whether this is similar in *Simkania* infection but could not detect any degradation of the BH3-only proteins Bad, Bid, Puma, Bim and Bmf (Figure S4). We also tested for the up-regulation of anti-apoptotic Bcl-2 family members Bcl-2 and Mcl-1, as the latter has previously been found to be up-regulated and stabilized in *C. trachomatis* infection [41], [42]. Interestingly, Mcl-1 and Bcl-2 levels remained constant in a time course infection experiment (Figure 5A), indicating that regulation of these Bcl-2 family members does not account for the apoptosis resistance in *Simkania* infection.

### Akt is activated and necessary for Simkania-induced apoptosis resistance

To further determine the origin of *Simkania*-mediated anti-apoptosis we tested the major survival pathway directly involved in TNFR signaling. We first tested activation of the serine/threonine kinase Akt that promotes survival by phosphorylating and inactivating pro-apoptotic molecules such as Ask1, Bad, Bax and FoxO3a [35]. In a time course infection experiment we observed that Akt was indeed strongly phosphorylated in infected cells (Figure 5A).

To test whether activation of Akt is required for apoptosis inhibition, inhibitor experiments were performed. The Akt pathway was investigated by blocking the upstream activator phosphatidylinositol 3-kinase (PI3K) using LY-294002 [53]. Treatment of infected HeLa cells (day 3 p.i., MOI 1) with LY-294002 before induction of apoptosis resulted in a marked dose-dependent apoptosis sensitization (Figure 5B–D) as judged from PARP cleavage and apoptotic cell count. These results show that the activity of Akt is required in *Simkania*-infected cells to resist apoptosis induction.

### cIAPs are up-regulated and required for apoptosis resistance in Sn-infected cells

**Figure 6:**
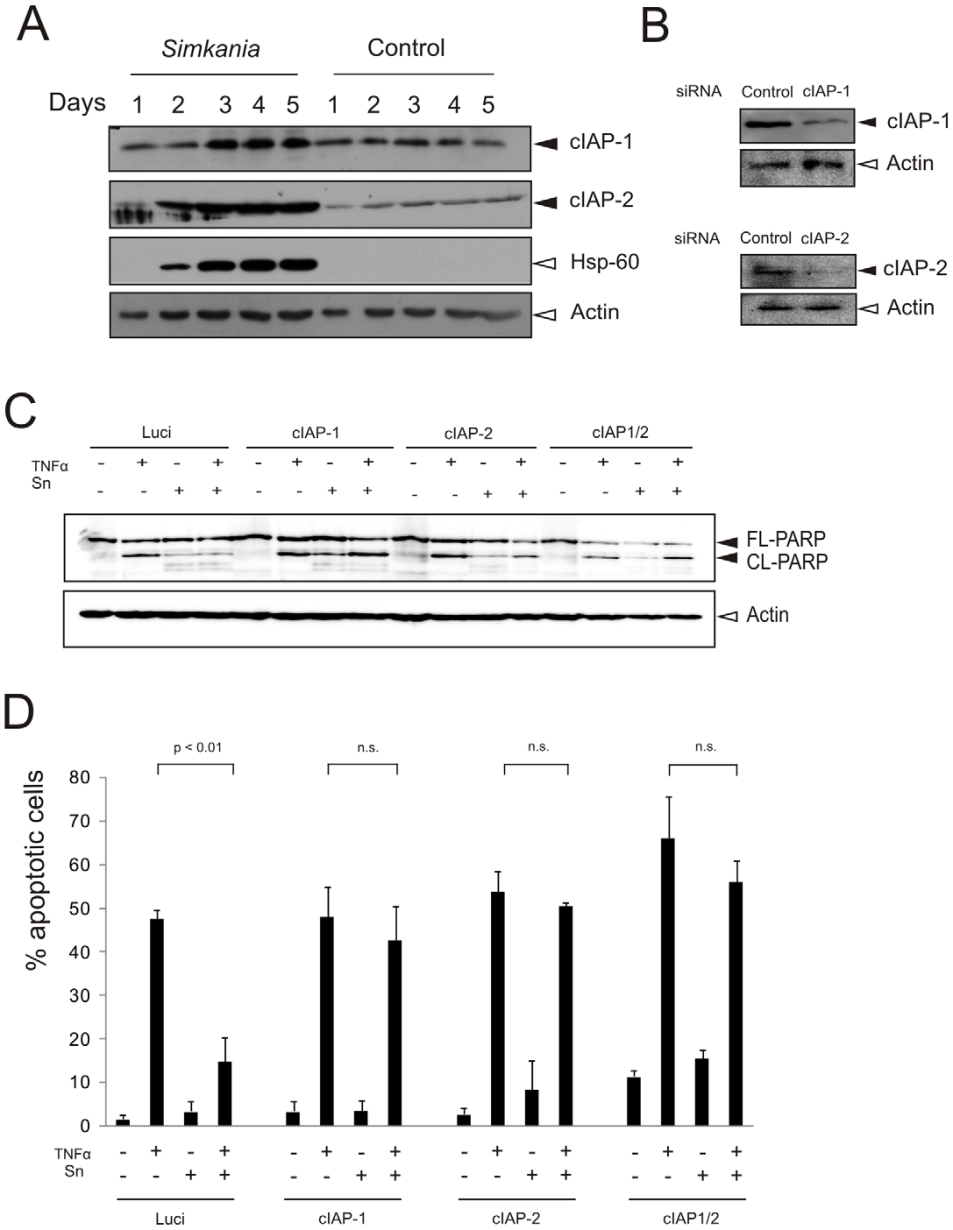
cIAPs are upregulated on *Simkania* infection and are required for the resistance of apoptosis. (**A**) Immunoblot analysis of the anti-apoptotic/pro-survival apoptosis regulators during S*imkania* infection. HeLa cells were infected with *Simkania* or Mock (control) (MOI 1) in a time course experiment before analysis. Anti-apoptotic cIAP-1 and -2 (black arrowheads) are strongly upregulated. Hsp-60 and Actin (white arrowheads) were used as loading controls. n = 2. (**B**) Immunoblot confirmation of cIAP-1 and -2 knockdown. HeLa cells were transfected with siRNA directed against cIAP-1, -2 or Luciferase (control). cIAP-1 and -2 (black arrowheads) were strongly down regulated at day 3 post transfection. Actin (white arrowheads) was used as loading control. n = 2. (**C**) Immunoblot analysis of PARP cleavage in S*imkania* infected cells after cIAP-1 and -2 single or cIAP-1/-2 double knockdown. HeLa 229 cells were transfected for 3 days before *Simkania* infection. On day 3 post infection cells were induced with 20 ng/ml TNF-α+3 µg/ml Chx or carrier for 4 hours before analysis. PARP cleavage (black arrowheads) was a measure of apoptosis sensitization. Single knockdown of either cIAP-1 or -2 or both clearly sensitized infected cells to apoptosis. Actin (white arrowhead) was used as loading control. n = 2. (**D**) Bar diagram showing samples treated similar as in Figure 6C but prepared for immunofluorescence analysis. Hoechst stained cells were counted to determine the number of apoptotic cells (40× magnification, five fields per sample, n = 2, Error bars = SE). Single knockdown of either cIAP-1 or -2 or both was found to restore apoptosis sensitivity to ∼70% of control.

We have previously reported that IAPs play a vital role in apoptosis inhibition of *C. pneumoniae*- and *C. trachomatis*-infected cells [43], [44]. Hence, we tested for the regulation of IAPs in Sn infected cells. Interestingly, we found that cIAP-1 and cIAP-2 were up-regulated in the course of infection (Figure 6A). We therefore tested if these IAPs are required to maintain the high apoptosis resistance in infected cells. Expression of cIAP-1 and cIAP-2 was silenced in control and infected cells using specific siRNAs and induced with TNF/Chx for apoptosis. The infected cells were found sensitized for apoptosis in the absence of cIAP-1 and cIAP-2 (Figure 6B–D), supporting their role in blocking apoptosis in infected cells.

## Discussion

Apoptosis resistance has been a common denominator as an outcome of infection with and a likely prerequisite for the development of obligate intracellular pathogenic *Chlamydia*. Here, we have demonstrated that the evolutionary related bacterium *Simkania negevensis* is also able to block host apoptosis. We provide evidence that anti-apoptotic and survival mechanisms are widely conserved over the order *Chlamydiales*. An exception to this rule may be *Parachlamydia* which appears to survive in human macrophages but eventually triggers apoptosis [22]. The inability to induce apoptosis inhibition may be one reason why *Parachlamydia* does not replicate in most mammalian cell types [54].

It is still unclear whether *Simkania* and *Parachlamydia* are able to influence death of *Amoebae*. Signaling to cell death inhibition initially could have co-evolved as a consequence of the *Simkania - Amoebae* interaction and could have later been exploited during the exposure to mammalian cells. Adaptation to different hosts may, at least in part, explain the differences we observed in anti-apoptotic signaling between cells infected with members of *Chlamydiales* adapted to *Amoebae* (*Simkania*) and mammalian cells (*C. trachomatis*, *C. pneumoniae*).

It has previously been suggested that apoptosis inhibition depends on the developmental cycle of *Chlamydia* with the onset early after EB to RB transition and maximum during RB replication [23]. The developmental cycle of *Simkania* lasts up to 10 days and therefore significantly longer than that of *C. trachomatis* (48 h) and *C. pneumoniae* (72–96 h) under comparable infection conditions. It was therefore unexpected to see that even at an MOI of 0.5, conditions, where one EB infects one cell, about 50% of the cells were protected from apoptotic stimuli already at 24 h p.i. and all infected cells were completely protected at three days p.i. A similar efficient protection at such low MOI has neither been observed with *C. trachomatis* [23] nor with *C. pneumoniae* [39], [40]. The infected cell remained resistant for apoptotic stimuli up to day 4 p.i. indicating that *Simkania* has evolved highly efficient and long-lasting mechanisms to block host cell death. Similar to *C. trachomatis* infection, cells infected at higher MOI acquired apoptosis resistance earlier (Figure 2C) which has been suggested to be dependent on the rapid entry of the bacteria into the replicative phase of the developmental cycle [23]. Interestingly, similar as with *C. trachomatis*, infection with *Simkania* at a higher MOI up to 100 had no visible cytotoxic effect (KK and TR, unpublished), whereas significant cytotoxicity already starting at an MOI of 10 has been reported for *C. pneumoniae* infection [40]. Different to what has been reported for *C. trachomatis* infected Mccoy cells, Sn did not induce apoptosis in neighboring uninfected cells during any stage of the infection, excluding paracrine apoptosis induction [21].

Despite differences in the kinetics of the onset, efficiency and duration of apoptosis resistance, all our data demonstrate a block in apoptotic signaling upstream or at the mitochondria. The mechanism underlying apoptosis resistance in *Simkania*-infected cells may thus be very similar to those in cells infected with *C. trachomatis* and *C. pneumoniae*, in which apoptotic signaling via mitochondria is prevented [38], [42]. Neither Bak nor Bax were found activated in Sn-infected cell treated with TNF/Chx and cytochrome *c* was fully retained in the mitochondria of these cells. Similar as we have found for *C. trachomatis*-infected cells [42], this complete and strong block of apoptotic signaling was maintained in the infected cell despite of unchanged levels of BH3-only proteins. In contrast, others have found degradation of BH3-only proteins in cells infected with *C. trachomatis* [45], [46], indicating that depletion of BH3-only proteins plays a role in blocking apoptosis upstream of the mitochondria in their infection setting.

We currently do not know how mitochondrial signaling is blocked in Sn-infected cells. Unlike in *C. trachomatis* infection [42], Mcl-1 as one of the major inhibitors of Bak and Bax activation was neither up-regulated nor stabilized in Sn-infected cells. Also the levels of Bcl-2 remained unchanged in the course of Sn infection (Figure 5A).

The data presented here and our previously published work demonstrates that IAPs play a major role in maintaining apoptosis resistance in cultured cells infected with *Simkania*, *C. trachomatis* and *C. pneumoniae* [43], [44]. However, recent data obtained in pulmonary infection experiments with *C. pneumoniae* in knockout mice suggest an important function of cIAP-1 in innate immune signaling in macrophages [55]. A role of IAPs in innate immune signaling is consistent with recent reports demonstrating a role of both, cIAP-1 and cIAP-2 in innate immune signaling rather than in directly inhibiting caspase activation [31]. Infection may directly influence IAP stability as these proteins possess E3 ligase activity and control their own stability and the stability of other IAPs [56]. In this context the previous description of IAP-IAP hetero complexes implemented in IAP stabilization in *C. trachomatis*-infected cells may also be relevant for *Simkania* infection [43].

As previously described for *C. trachomatis* [47] and *C. pneumoniae* [57] infection, *Simkania* infection strongly activates the PI3K-Akt pathway. Activated Akt promotes cell survival through crosstalk with other signaling cascades like the NF-κB pathway [58]. Akt has in addition a role in cell survival by increasing the uptake of nutrients, positive regulation of metabolic pathways and maintenance of mitochondrial membrane potential [59]. There is also a direct link between Akt and cell survival by phosphorylating and inhibiting the function of pro-apoptotic Bad [60]. We could not observe, however, any changes in the Akt-specific phosphorylation of sites S116 and S136 in Bad in the course of *Simkania* infection (KK and TR, unpublished), as has previously been described for *C. trachomatis* infection [47]. The observation that PI3K inhibitors sensitize cells infected with *C. trachomatis* [42] or *Simkania* to apoptosis speaks in favor for an evolutionary conserved role of this central survival pathway in controlling host cell survival during development and replication of *Chlamydiales* in their mammalian hosts.

Activation of Akt can favor high expression of cIAP-1 [61], consistent with elevated levels of IAPs and increased phosphorylation of Akt during the course of infection until day 5. This raises the questions how these long-term signaling events are fueled even several days after the first interaction of the pathogen with the host cell. One could speculate that the bacterial factors play a crucial role in manipulating the host. Like other pathogenic members of the *Chlamydiales*, *Simkania* has been reported to have a type III secretion system which may help to transport proteins across the inclusion membrane into the cytosol of the host [62]. Secreted bacterial proteins may take over the pacemaker function in maintaining the host cell in a survival mode to guarantee such a long term host pathogen relation.

## Materials and Methods

### Cell culture, propagation of Simkania negevensis

HeLa 229 cells (CCL-2.1) obtained from ATCC were grown in DMEM supplemented with 10% fetal calf serum (FCS). For propagation of *Simkania*, HeLa 229 cells were grown in DMEM medium supplemented with 5% FCS, 2 mM L-glutamine, 10000 U/ml penicillin, 10 mg/ml streptomycin, 8 µg/ml gentamicin and 50 µg/ml vancomycin [63] and 1 µg/ml Chx. Bacterial purification was done as described [64] with the exception that *Simkania* was harvested on day 5 post infection.

### Antibodies, counter stains and chemicals

Chemicals were obtained from Sigma-Aldrich unless otherwise indicated. Rabbit polyclonal anti-human active Caspase-3, Caspase-9, Bid, ERK, MEK, p-ERK and p-MEK were from Cell Signaling Technology. Mouse monoclonal anti-human PARP, cytochrome *c*, cIAP-2, cIAP-1, BAX (clone 6A7) and mouse monoclonal anti-*Chlamydia* Hsp60 were purchased from Santa Cruz Technology. Mouse monoclonal anti-human β-Actin was from Sigma Aldrich. Mouse anti-human BAK (Ab-1) was obtained from Oncogene Research Products. Secondary antibodies were horseradish peroxidase-labelled rabbit anti-mouse or anti-rabbit antibodies from Amersham Biosciencesor Cy2, Cy3 or Cy5 - labeled anti-mouse or anti rabbit antibodies from Dianova, Immunodiagnostic. Syto82, Draq5 and Hoechst 33258 were obtained from Invitrogen (Molecular probes), Alexis and Sigma-Aldrich, respectively.

### Apoptosis induction

Infected and control cells were induced for apoptosis with 20 ng/ml of human recombinant TNF-α (BD Pharmingen, San Diego, California) with 3 µg/ml of Chx for 4 h or with 1 µM STS for 5 h. The control cells were incubated with Chx. After the respective time post induction, the stimulated and control cells were fixed with 4% paraformaldehyde for 20 min for immunostaining or harvested using lysis buffer (4% SDS, 20% glycerol, 10% 2-mercaptoethanol, 0.004% bromphenol blue, 0.125 M Tris HCl) and analyzed by western blotting.

### Immunofluorescence analysis

For immunofluorescence analysis, cells were seeded on glass coverslips (Marienfeld Laboratory glassware, Germany) of 15 mm diameter and then infected or treated as uninfected controls. The cells were treated appropriately for the concerned experiment and washed in phosphate-buffered saline (PBS) and fixed with 4% paraformaldehyde in PBS for 20 min. After washing, the cells were permeabilized using 0.2% Triton-X100 in PBS for 20 min and blocked with 2% FCS in PBS for 45 min. Cells were incubated for 1 hour with the respective primary antibody at 1∶50–1∶100 dilution in blocking buffer (2% FCS in PBS), washed three times in PBS, and stained with the corresponding secondary antibody. For counter staining cells were treated with Hoechst 33258 (5 µg/ml) or Draq5 for 30 min at room temperature to detect nuclei and intracellular bacteria. After washing three times with PBS, samples were mounted onto slides using Mowiol 4–88 (Carl Roth, Germany). Samples were analyzed on either a Leica DMR epifluorescence or Leica SPE confocal microscope. For apoptosis assays cells from five random 40× magnification fields were counted. The percentage of apoptotic cells was calculated as number of apoptotic cells/number of total cells. Terminal deoxynucleotidyltransferase-mediated nick-end labelling *(TUNEL) assays* were performed according to manufacturer's instructions (Promega, Madison.) and were analyzed as previously described [64].

### Detection of Caspase-8 activation

HeLa 229 cells were seeded in 96 well plates at a concentration of 15000 cells/ml. Cells were infected with Sn and after 3 days of infection the cells were apoptosis induced with TNF/Chx as described above. The assay was performed using caspase glo-8-assay (Promega) according to the manufacturer's instructions and was analyzed using a Infinite M200 multiplate reader (Tecan).

### Immunoblotting and in vitro cross-linking of proteins

Cell monolayers were collected and lysed with sample buffer (3% 2-mercaptoethanol, 20% glycerine, 0.05% bromophenol blue and 3% SDS) and boiled at 90°C for 5 min. Immunoblotting was performed as previously described [64]. For *in vitro* cross-linking of proteins, HeLa 229 cells were grown in 35 cm^2^ plates to 60–70% confluency. Apoptosis was induced as described in the apoptosis induction section or mock induced in the presence of 3 µg/ml Chx. Supernatant was collected and the remaining cells were scraped with a rubber police and washed in PBS by centrifugation at 1000 rpm for 3 min at 4°C. For mitochondrial isolation, the pellet was resuspended in Buffer A (20 mM Hepes pH 7.6, 220 mM mannitol, 70 mM sucrose, 1 mM EDTA, 0.5 mM PMSF and 2 mg/ml BSA) and left on ice for 15 min. Cells were lysed with a homogenizer and pelleted at 800 g for 5 min at 4°C. Mitochondria containing supernatant was collected and centrifuged again at 10.000 g for 10 min at 4°C. Mitochondrial pellet was resuspended in Buffer B (Buffer A without BSA), quantified using a Nanodrop (Peqlab, Germany) and stored at −80°C. Supernatant containing cytoplasmic proteins was kept in cytochrome *c* release assays. 60 µg mitochondria were pelleted (10.000 g, 10 min, 4°C) and washed once with SET-buffer (250 mM sucrose, 10 mM Tris/Hcl pH 7.6, 1 mM EDTA) and resupended in 95 µl SET-buffer. Cross linking was done with BMH (Pierce, Rockford) at a final concentration of 1 mM for 30 min at room temperature. For quenching of the cross linker 2 µl DTT (2.5 mM) were added and samples were incubated for 5–10 min on ice. Cross linked mitochondria were pelleted (14.000 rpm, 5 min, 4°C) and washed once in SET-buffer, resuspended in 30 µl Laemmli-buffer and analyzed by western blotting.

### siRNA transfection

HeLa 229 cells were seeded in 12-well plates one day before transfection. siRNA was transfected using Lipofectamine 2000 (Invitrogen) according to manufacturer's instructions. Six hours post transfection medium was exchanged and 24 hours post transfection cells were infected with Sn (MOI 1) for 3 days before apoptosis induction with TNF-α. Knockdown was confirmed via immunoblotting. The following siRNAs were used in the study: siLuc (Control): *AACUUACGCUGAGUACUUCGA* , sicIAP-1: *AACAUAGUAGCUUGUUCAGUG* , sicIAP-1 *CUAGGAGACAGUCCUAUUCAA* , sicIAP-2: *AAUUGGGAACCGAAGGAUAAU* , sicIAP-2 TTCAAGAUACACAGUUU CUAA. The respective two siRNAs were used as a pool.

### Statistical analysis

For statistical calculations and histograms Excel (Microsoft) was used. Statistical significance was calculated using the students T test.

## Supporting Information

Figure S1 **Sn infected cells block the TNF-α induced apoptosis in HeLa cells.** HeLa cells with or without *Simkania* infection (MOI 1) were treated with 20 ng/ml TNF-α+3 µg/ml Chx or with carrier for 4 hours. Samples were stained with Hoechst (blue) and viewed under a fluorescent microscope. Hoechst dye stained both HeLa cell nuclei and *Simkania* inclusions. The infected cells are marked by white asterisk. Uninfected cells show apoptotic cells which are bright spots due to nuclear condensation. Infected induced cells show a clear block of apoptosis. n = 2.
(TIF)

Figure S2 **TNF receptor is activated in** ***Simkania*** **infected cells.** HeLa cells with or without *Simkania* infection (MOI 1) were treated with 20 ng/ml TNF-α without Chx for 5, 15, 30 and 60 minutes to activate the MEK-ERK pathway. The figure shows the phosphorylation of MEK (grey arrowheads) and ERK (white arrowheads), indicating that the TNF receptor is active. Actin is used as the loading control. n = 2.
(TIF)

Figure S3 **Sn infected cells block the activation of caspase-3.** Bar diagram displaying the quantitative analysis of the caspase-3 activation at MOI 0.5 from the experiment shown in figure 3B. Cells from five random fields were counted under a 40× objective and the percentage of caspase-3 positive cells was calculated. *Simkania* infection strongly reduced caspase-3 activation. n = 3, error bars = SE.
(TIF)

Figure S4 **Pro-apoptotic BH3 only Bcl-2 family members are not regulated.** Immunoblot analysis of the pro-apoptotic BH3 only Bcl-2 family members during S*imkania* infection. HeLa cells were infected with *Simkania* or Mock (control) (MOI 1) in a time course experiment before analysis. The BH3 only proteins (Bad, Bid, Puma, Bim, Bmf, black arrowheads) are not regulated in *Simkania* infected cells. Hsp-60 and Actin (white arrowheads) were used as loading controls. n = 2.
(TIF)
