# Supplementary figures and images for: Evolutionary Conservation of Infection-Induced Cell Death Inhibition among Chlamydiales
Source: PLoS One. 2011 Jul 22;6(7):e22528. doi: 10.1371/journal.pone.0022528 (PMC3142178; doi:10.1371/journal.pone.0022528)

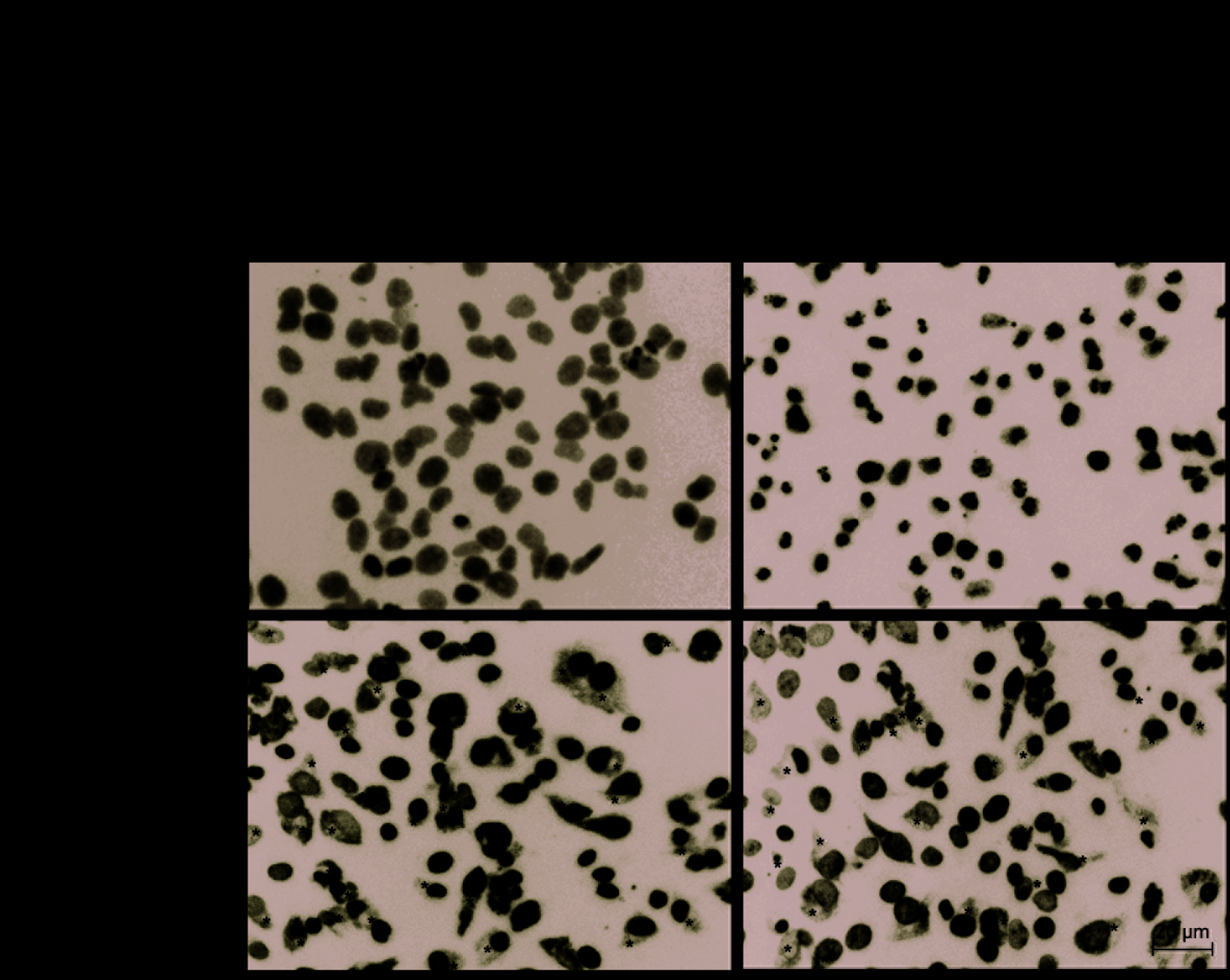

Supplement: Figure S1 — Sn infected cells block the TNF-α induced apoptosis in HeLa cells. HeLa cells with or without Simkania infection (MOI 1) were treated with 20 ng/ml TNF-α+3 µg/ml Chx or with carrier for 4 hours. Samples were stained with Hoechst (blue) and viewed under a fluorescent microscope. Hoechst dye stained both HeLa cell nuclei and Simkania inclusions. The infected cells are marked by white asterisk. Uninfected cells show apoptotic cells which are bright spots due to nuclear condensation. Infected induced cells show a clear block of apoptosis. n = 2. (TIF) [file pone.0022528.s001.tif]

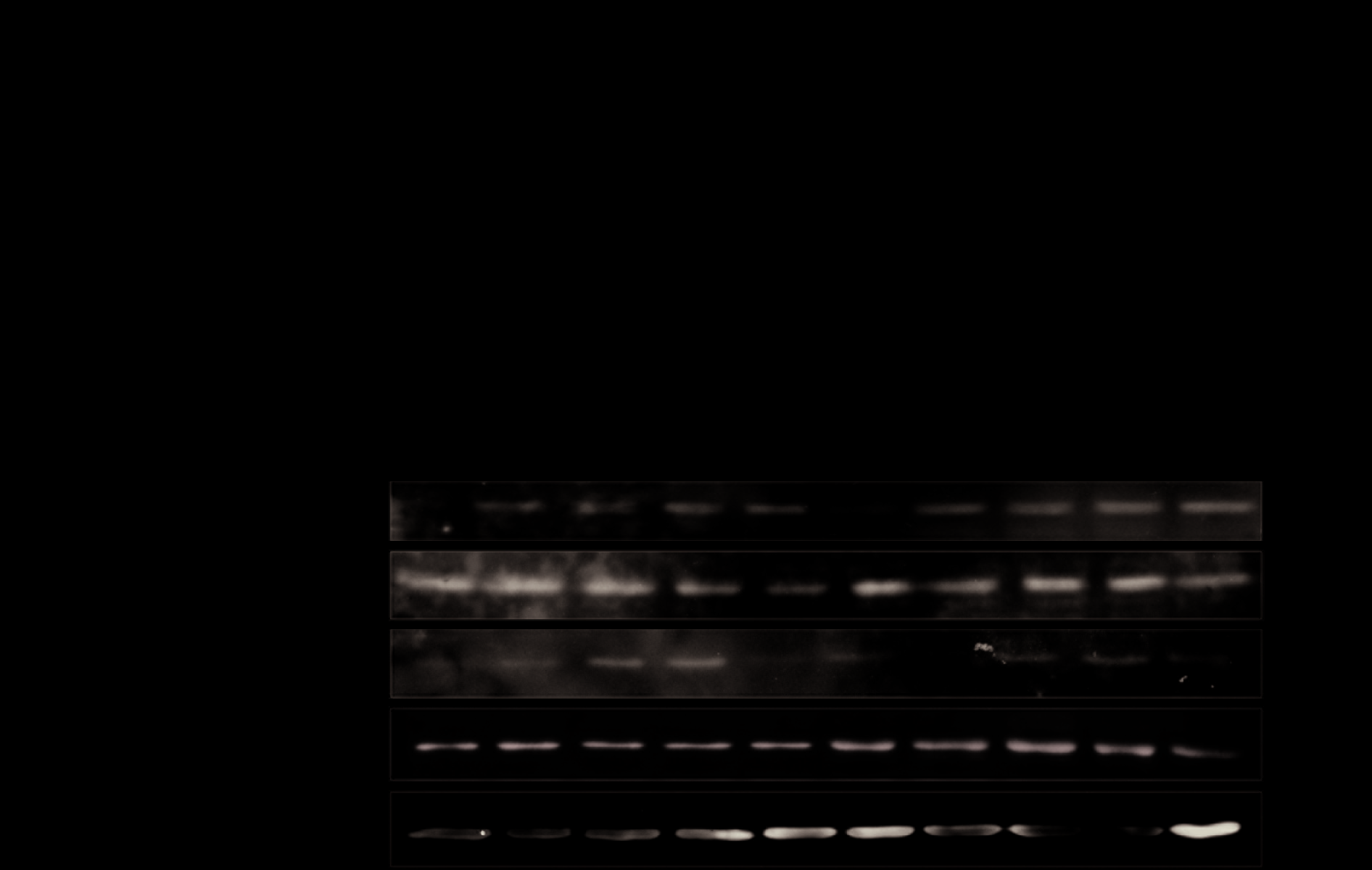

Supplement: Figure S2 — TNF receptor is activated in Simkania infected cells. HeLa cells with or without Simkania infection (MOI 1) were treated with 20 ng/ml TNF-α without Chx for 5, 15, 30 and 60 minutes to activate the MEK-ERK pathway. The figure shows the phosphorylation of MEK (grey arrowheads) and ERK (white arrowheads), indicating that the TNF receptor is active. Actin is used as the loading control. n = 2. (TIF) [file pone.0022528.s002.tif]

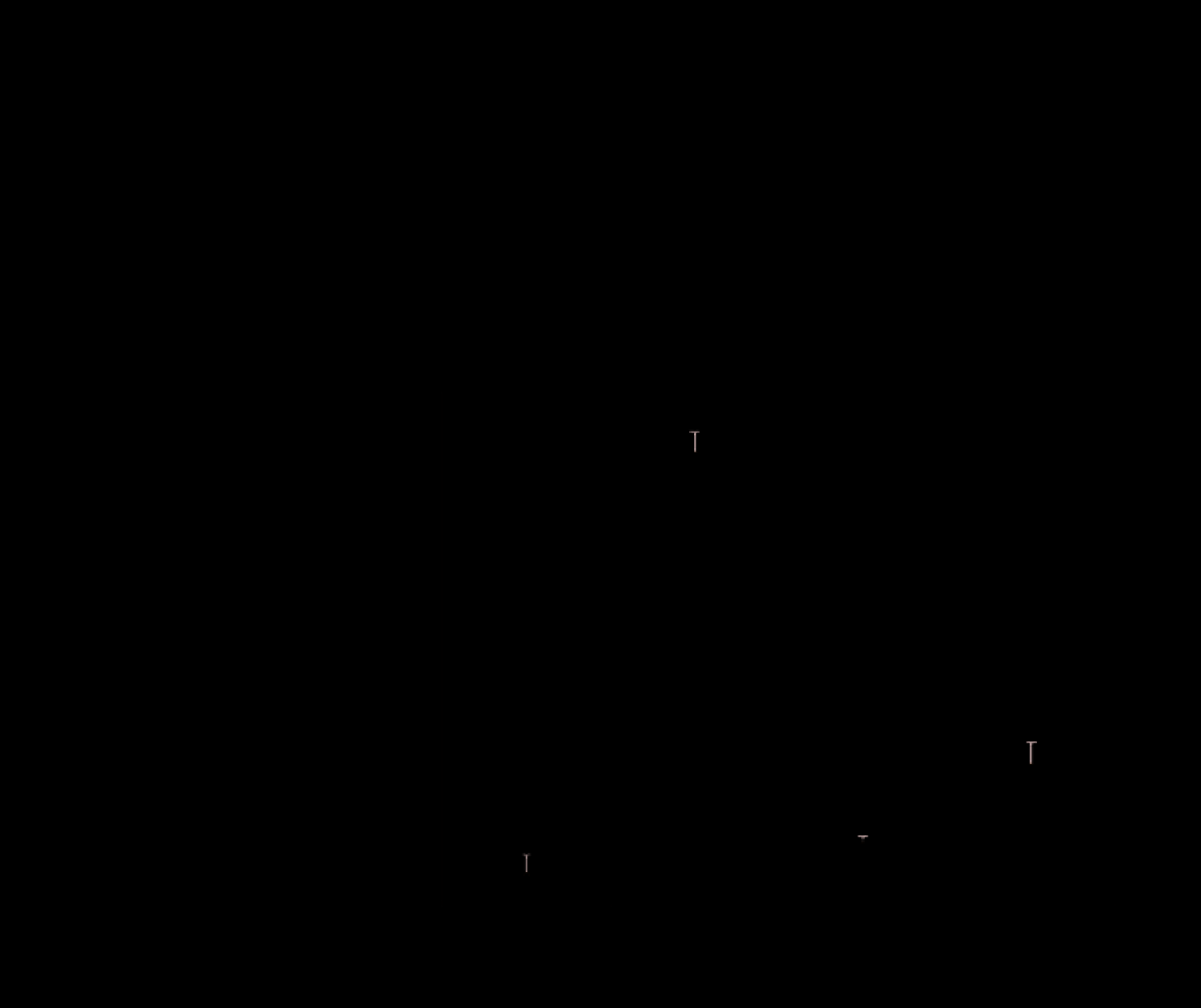

Supplement: Figure S3 — Sn infected cells block the activation of caspase-3. Bar diagram displaying the quantitative analysis of the caspase-3 activation at MOI 0.5 from the experiment shown in figure 3B. Cells from five random fields were counted under a 40× objective and the percentage of caspase-3 positive cells was calculated. Simkania infection strongly reduced caspase-3 activation. n = 3, error bars = SE. (TIF) [file pone.0022528.s003.tif]

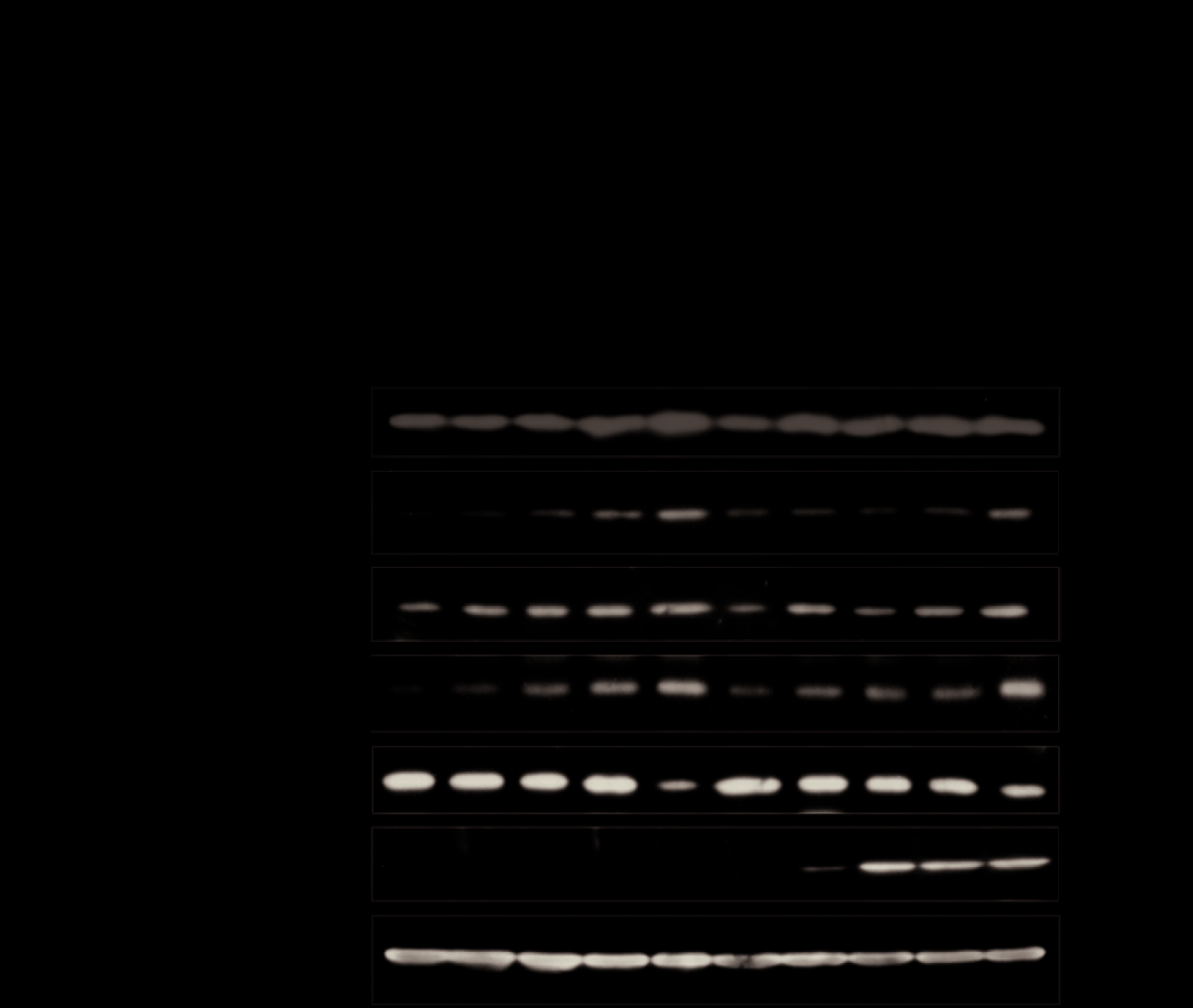

Supplement: Figure S4 — Pro-apoptotic BH3 only Bcl-2 family members are not regulated. Immunoblot analysis of the pro-apoptotic BH3 only Bcl-2 family members during Simkania infection. HeLa cells were infected with Simkania or Mock (control) (MOI 1) in a time course experiment before analysis. The BH3 only proteins (Bad, Bid, Puma, Bim, Bmf, black arrowheads) are not regulated in Simkania infected cells. Hsp-60 and Actin (white arrowheads) were used as loading controls. n = 2. (TIF) [file pone.0022528.s004.tif]
